# Pregnancy reporting and biases in under-five mortality in three African HDSSs

**DOI:** 10.1080/00324728.2025.2573925

**Published:** 2025-11-12

**Authors:** Hallie Eilerts-Spinelli, Julio E. Romero-Prieto, Kobus Herbst, Dickman Gareta, Momodou Jasseh, Sammy Khagayi, David Obor, Jeffrey W. Imai-Eaton, Georges Reniers

**Affiliations:** 1 London School of Hygiene and Tropical Medicine,; 2 Johns Hopkins University,; 3 Africa Health Research Institute,; 4 DSTI-SAMRC South African Population Research Infrastructure Network,; 5 MRC Unit The Gambia at LSHTM,; 6 Kenya Medical Research Institute,; 7 Harvard T.H. Chan School of Public Health,; 8 Imperial College London

**Keywords:** HDSS, pregnancy registration, pregnancy surveillance, pregnancy reporting, under-five mortality, child mortality, postneonatal mortality, neonatal mortality, measurement error, sub-Saharan Africa

## Abstract

In the absence of complete civil registration and vital statistics, Health and Demographic Surveillance Systems (HDSSs) are important sources of population-based data throughout sub-Saharan Africa. However, HDSS data on the vital status of newborns are often unreliable due to omission of those who were born and died between two rounds of data collection and are therefore never enumerated. This study investigates whether pregnancy registration improves estimation of under-five mortality (U5M) in three HDSSs in The Gambia, Kenya, and South Africa. We find that mortality is higher for children whose mother’s pregnancy was observed than for children who were first registered after birth. Cox proportional hazards models with inverse probability weights further suggest that this difference is probably due to improved ascertainment of deaths in pregnancy cohorts and unlikely to be driven by a selection effect. These results highlight the importance of pregnancy registration in HDSSs for the estimation of U5M.

## Introduction

In 2022 an estimated 2.8 million African children aged under five died from causes that are mostly preventable (UN IGME 2024). Accurate measurement of levels, trends, and age patterns of under-five mortality (U5M) is essential for tracking and accelerating progress towards its reduction. Civil registration and vital statistics (CRVS) systems are the preferred sources for this information, generating reliable and up-to-date data on births, deaths, and causes of death (AbouZahr et al. 2015). Unfortunately, national CRVS systems are either incomplete or non-existent in regions with the highest U5M burden, including most of sub-Saharan Africa (Mikkelsen et al. 2015). Retrospective maternity history questionnaires included in sample surveys (e.g. Demographic and Health Surveys [DHS], Multiple Indicator Cluster Surveys [MICS]) often serve as the foundation for U5M estimates in such settings. The recent termination of the DHS Program represents a major loss for global health monitoring (Khaki et al. 2025). And while the absence of these surveys constitutes a far greater challenge, it is also important to acknowledge that retrospective maternity histories are subject to well-documented retrospective reporting errors, which can introduce bias into U5M estimates (Pullum and Becker 2014). Health and Demographic Surveillance Systems (HDSSs) are another source of population-based data: more valuable now than ever and standing apart for collecting detailed epidemiological and socio-demographic information on a longitudinal basis.

HDSSs conduct surveillance on geographically defined populations of several thousand individuals. After an initial census, information is typically collected through regular household interviews spaced between every three months and one year apart, depending on the study. Data collected on births and deaths in HDSSs are less subject to recall bias than those from retrospective surveys, as interviewees are asked to report only on events occurring since the last interview round. Once individuals are added to a household roster, they are followed up at each subsequent data collection visit, a process that generates rich longitudinal data. HDSSs are often considered the gold standard for demographic, health, and social dynamics data in areas of sub-Saharan Africa where other high-quality data are not available.

While the prospective data collection in HDSSs is highly effective at tracking the vital status of established residents, reporting on new and transient residents is less exhaustive. This limitation applies especially to babies who are born and die between two rounds of data collection. Those who survive to the first HDSS interview following their birth are more likely to be identified and included on the household roster. This facilitates the tracking of all their future events such as mortality or out-migration. However, early deaths (i.e. those taking place in the first few months of life) are frequently missed, either because the respondent does not report the birth and death or as a result of insufficient probing by the interviewers. Therefore, there are persistent concerns regarding the accuracy of HDSS data collected on the mortality of children aged under five, especially within the first few weeks of life, where the risk of mortality is highest. Under-reporting of early deaths is an issue that plagues survey-based estimates of U5M too (Pullum and Becker 2014). However, the prospective design of HDSS data collection makes this source well positioned for assessing and addressing such biases.

This work investigates downward bias in HDSS estimates of early mortality, using pregnancy data from Basse HDSS (The Gambia), Siaya HDSS (Kenya), and Somkhele HDSS (South Africa). These sites all practise pregnancy surveillance, where pregnancies of women of reproductive age are registered during routine data collection and followed up in subsequent rounds for pregnancy outcomes. We begin with a descriptive analysis of pregnancy registration at each site. We then compare U5M estimates for *pregnancy cohorts* (i.e. where registration occurred during pregnancy) and *birth cohorts* (i.e. where registration first took place after birth). U5M estimates for births with prior pregnancy registration or counting exposure time since birth only if the pregnancy was registered have previously been found to be *higher* than estimates for the entire HDSS cohort (Cantrelle 1969; Nareeba et al. 2021). However, what is not clear from the existing literature is whether this finding is due to improved ascertainment of early deaths following pregnancy registration or due to selection bias from shared risk factors for pregnancy registration and U5M. To answer this question, we use micro-level analysis to investigate aggregate differences in the observed mortality of pregnancy and birth cohorts.

Our approach uses inverse probability weighting for pregnancy registration in Cox proportional hazards models for U5M; this is a method that can be used for evaluating selection bias. Weights are used to standardize cohorts with respect to observable characteristics, effectively creating a pseudo population in which pregnancy registration is independent from all covariates and its marginal effect on mortality measurement can be estimated. Inverse probability weighting has long been used in survival analysis to estimate effects of non-randomized treatments (Robins et al. 2000; Hernán et al. 2001). We present a novel application of these methods to pregnancy registration in HDSSs and find that the higher mortality of pregnancy cohorts is not attributable to negative selection but rather to improved capture of early deaths. We recommend that pregnancy surveillance be prioritized in HDSSs to improve the accuracy of early mortality estimates.

## Background

### Downward bias in HDSS estimates of early childhood mortality

Under-registration of early deaths has long been documented in surveillance-based research in sub-Saharan Africa. Longitudinal cohort studies collecting mortality and morbidity data in West Africa in the 1950s and ‘60s noted the tendency for deaths occurring shortly after birth to be undercounted (Cantrelle 1969; Billewicz and McGregor 1981). Certain studies collected information on pregnancies to reduce the risk of omissions (Cantrelle and Leridon 1971; Cantrelle 1974), although such data were incomplete and downward bias likely persisted (Garenne 1981; Pison and Langaney 1985). These early surveillance studies paved the way for other population health research stations to be founded across the continent in the 1980s and ‘90s (Ngom 2001). Over time, demographic surveillance practices have become more standardized as independent research centres engaged in networks of affiliated HDSSs (Utazi et al. 2018). HDSSs throughout sub-Saharan Africa generate robust prospective data on a wide array of population health indicators. Nevertheless, measurement of early mortality remains a challenge and is a regularly reported weakness in HDSS data (Kahn et al. 2012; Rossier et al. 2012; Sankoh and Byass 2012; Kishamawe et al. 2015; Assefa et al. 2016).

Under-reporting can be due to a variety of factors. Children who are born and die between data collection rounds can easily be missed because their identification requires careful probing by fieldworkers. Furthermore, data collection is sometimes conducted using proxy respondents, who may not be as accurate in reporting on pregnancies and pregnancy outcomes as the women themselves. Respondents may wish to avoid the stigma associated with the death of a child or discussion of an event that is painful to recall (Waiswa et al. 2019; Kambugyiro 2023). In settings with long-running surveillance systems, respondents may also be aware that reporting a death will trigger additional follow-up questions and thus omit the event due to interview fatigue (Hinga et al. 2021; Kambugyiro 2023). Late pregnancy and the postpartum period are also often times of increased mobility, when women travel to seek medical care or the support of family members (Clouse et al. 2018). Births and deaths occurring outside the HDSS site may be less likely to be reported on the mother’s return.

Huge variation in HDSS estimates of neonatal mortality for given levels of U5M is indicative of data-quality issues. In an analysis of data from 31 HDSS sites for the period 2009–14, Waiswa et al. (2019) found that four sites reported neonatal mortality rates of <10 deaths per 1,000 live births, whereas the highest estimate was 41.6 deaths per 1,000 live births. The interquartile range of such estimates was more than double that of national DHS estimates. A systematic comparison with large cross-sectional survey estimates from 1990 to 2018 noted important heterogeneity in HDSS estimates of neonatal mortality, which were on average 8 per cent lower than DHS estimates for subnational region and 14 per cent lower than MICS estimates (Eilerts et al. 2021). Given the differences in methods, sample compositions, and data collection schedules, some divergence of HDSS and survey estimates would be expected. However, both studies found that markers of good data quality, such as frequent interview rounds and precise date reporting, were linked to higher mortality estimates in HDSSs (Waiswa et al. 2019; Eilerts et al. 2021).

Extremely low levels of early mortality in HDSSs can also be considered implausible in light of established regularities in the age pattern of mortality. For instance, in the same analysis of data for 31 HDSS sites in 2009–14, the overall proportion of neonatal deaths occurring on the first day of life was only 1.3 per cent, compared with the expected level of around 40 per cent (Waiswa et al. 2019). Traditional mortality models are based mostly on the experience of European countries, and there is much evidence for the existence of a unique African age pattern of U5M (Cantrelle and Leridon 1971; Garenne 1981; Blacker et al. 1985; Pison and Langaney 1985; Jasseh 2003). However, a recent study found that while African HDSS and DHS estimates both showed excess mortality compared with expected patterns after six months of age, HDSSs were alone in their lack of excess mortality before 28 days of age (Verhulst et al. 2022). This disagreement between sources suggests issues of data quality and possible under-registration of early deaths in HDSSs.

### Pregnancy registration and follow-up

Demographic surveillance entails collection of data on the vital events of births, deaths, and migrations. Some HDSSs also record whether household members are pregnant at the time of the data collection visit, despite pregnancy not typically being defined as a vital event. These records, commonly referred to as pregnancy registrations or notifications, are stored in the HDSS database and, at certain sites, fed back into data collection tools so that HDSS fieldworkers are prompted to probe for the pregnancy outcome in subsequent data collection rounds. This follow-up process is key to distinguishing active pregnancy surveillance from the simple registration or notification of pregnancies. Pregnancy surveillance can transform the process of reporting recent births from retrospective to prospective, leveraging the strengths of HDSSs. However, the collection and use of pregnancy data in HDSSs is varied (Waiswa et al. 2019; Kwon et al. 2021). Protocols for pregnancy surveillance are less standardized across sites than those for other core components. For instance, some HDSSs record information on completed pregnancies when locating a live baby in routine data collection or impute pregnancy notifications for data-integrity purposes (Waiswa et al. 2019). At other sites, information on ongoing pregnancies is collected through pregnancy status reports but not integrated into subsequent data collection. Such systems allow for the collection of various pregnancy-related indicators but do not facilitate the follow-up of pregnancy outcomes.

Collecting data on pregnancies in HDSSs is challenging for a variety of reasons. Most sites use systems of proxy reporting, where one individual reports information on behalf of all household members. The use of a proxy respondent is necessary for reporting on a resident who has recently died or out-migrated. However, proxy reports are likely less effective for collecting information on pregnancies. Information regarding pregnancy status is sensitive, and women may conceal their pregnancy so as to avoid gossip, shame that can accompany giving birth outside marriage, or stigma associated with pregnancy loss (Haws et al. 2010; Kwesiga et al. 2021). In many cases, the proxy respondent may not be aware of the pregnancy status of women in their own household until it is evident. Whether or not the pregnancy is reported also depends on the frequency of HDSS data collection rounds. In sites where data collection rounds occur only once or twice per year, there is a low chance of the household interview coinciding with the later stages of gestation, when the pregnancy is most likely to be observed by the fieldworker or reported by or on behalf of the subject.

There are several other barriers to reporting pregnancies in HDSSs. The use of male interviewers negatively impacts data quality and completeness (Johnson et al. 2009; Harling et al. 2018), especially for such subjects as pregnancy and childbirth (Kadobera et al. 2017; Akuze et al. 2020). Some HDSSs employ female interviewers to collect data from women about pregnancy and childbirth, although recruiting and retaining staff can be a challenge (Alabi et al. 2014; Akuze et al. 2016). Other procedures, such as conducting the interview in a private place or engaging in rapport building to make the respondent feel comfortable, are essential for collecting data on sensitive events but not always included in HDSS interviewer training (Kwesiga et al. 2021). HDSS data collection protocols have been designed to accommodate diverse research priorities under strict organizational and resource constraints. In general, they are not specifically orientated towards collecting data on pregnancies, and incomplete pregnancy registration and outcome follow-up is often the result.

Despite cross-site heterogeneity in data collection protocols and substantial barriers to reporting, pregnancy registration could be key to addressing the quality issues in HDSS estimates of U5M. We investigate these issues in a comparative analysis of pregnancy reporting and U5M in three African HDSSs. This work contributes to understanding pregnancy registration as an integral component of HDSS data collection and one that has profound implications for the quality of early child mortality estimates.

## Data

**Figure 1:**
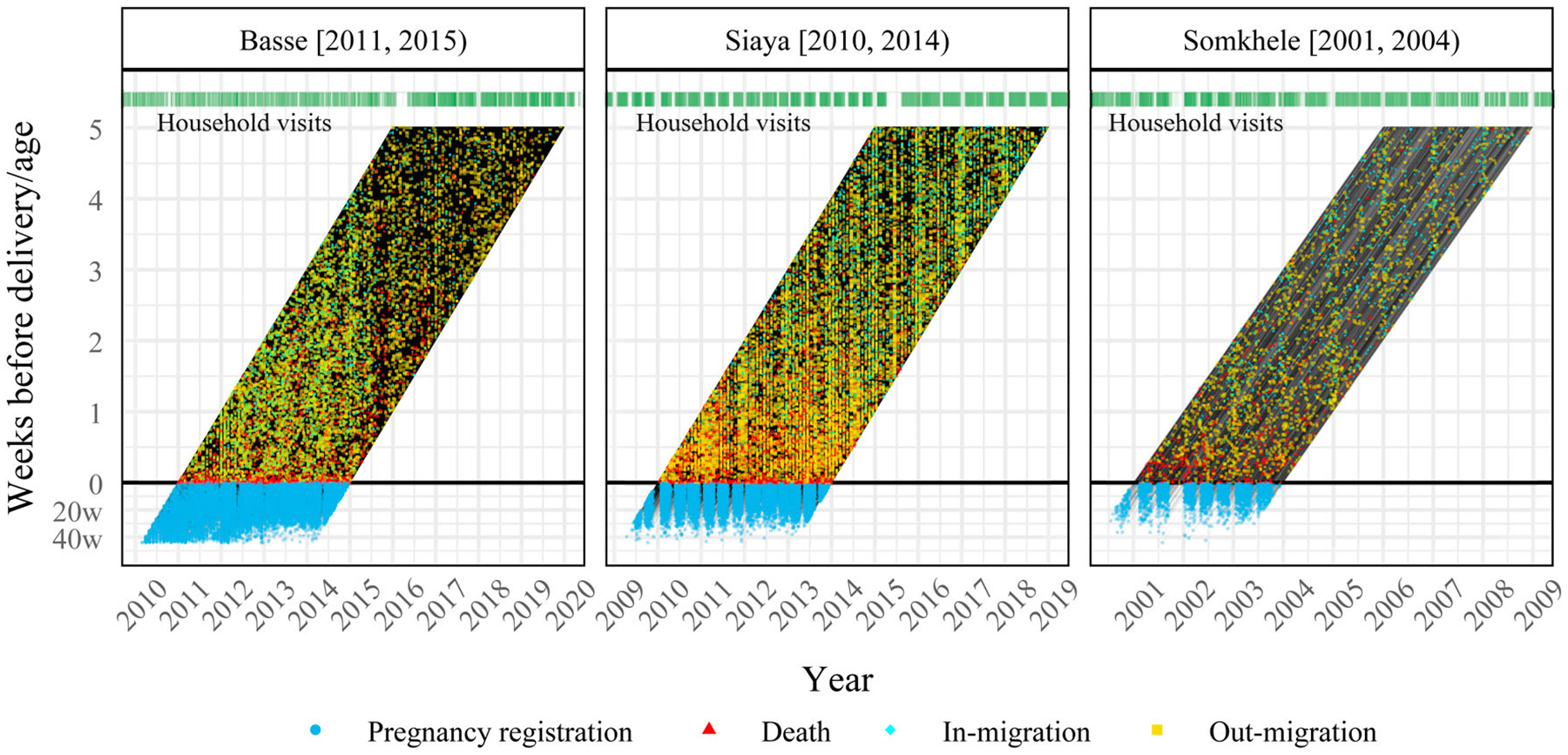
Lexis diagrams depicting the cohorts of children included in the analysis for each HDSS site *Notes*: The dates of data collection through regular household visits are plotted in bars across the top of each panel, with colour intensity denoting the quantity of data collected during the same day of fieldwork. In Basse the dates of data entry were used to infer household visits, which were not included in the data set. This figure is best viewed online in colour. *Source*: Authors’ own.

The analysis was conducted on cohorts of births taking place over three to four years at HDSS sites in Basse (The Gambia), Siaya (Kenya), and Somkhele (South Africa). In all three HDSSs, the nature of data collection has evolved over time with respect to the frequency of interview rounds, the size of the study area, and the availability of resources. Our analytical cohorts cover years where such factors were consistent throughout to the greatest extent possible. The quality of the data was also considered with a thorough evaluation of the precision and completeness of reported dates of pregnancies, births, and deaths and information on other socio-demographic variables. Periods where the number of pregnancies and births observed in the sites were relatively constant were prioritized, as were periods where pregnancy and birth cohorts were of roughly equal size. Finally, it was necessary to have at least five full years of follow-up time for babies born into the cohort, thus births taking place less than five years before the most recent data collection round were not eligible for inclusion. Figure 1 displays Lexis diagrams of the cohorts included in the analysis for each HDSS, with points plotted to denote the pregnancy registrations, deaths, out-migrations, and in-migrations of each subject.

### HDSS sites

#### Basse HDSS.

The Basse HDSS is run by the Medical Research Council (MRC) Unit The Gambia at the London School of Hygiene and Tropical Medicine (LSHTM). The town of Basse is on the south bank of the River Gambia, in the eastern Upper River region of The Gambia. The HDSS site is located in the Fulladu East and Kantora districts, in a predominantly rural setting. Demographic surveillance began in July 2007 to support ongoing studies related to pneumococcal and diarrhoeal diseases in infants and young children (UKRI n.d.). The prevalence of communicable diseases among the population is high. Malaria prevention measures have contributed to declining rates of infant and under-five mortality and an increasing concentration of deaths at neonatal ages (Jasseh et al. 2015).

The current analysis is based on 29,447 births that took place at the site between 2011 and 2015. During this time, the HDSS collected data in interview rounds occurring three times per year (Rerimoi et al. 2019). Resident village reporters were also tasked with keeping track of demographic events on an ongoing basis, and their records were used by HDSS fieldworkers to cross-check data collected during household interviews (Rerimoi et al. 2019). Interviews were conducted with heads of household (or suitable representatives), who reported events on behalf of all household members. Pregnancies that were reported in household interviews were followed up to record their outcome. In cases where the pregnancy was not registered, it was the practice of the Basse HDSS to impute a pregnancy report for the day before the birth.

#### Siaya HDSS.

The HDSS in Siaya County is located to the north-east of Lake Victoria in Nyanza Province, western Kenya. It is operated jointly by the Kenya Medical Research Institute (KEMRI) and the Centers for Disease Control and Prevention (CDC). In the early 1990s, the KEMRI–CDC partnership established surveillance infrastructure to collect information on malaria morbidity, mortality, and interventions as part of a trial of insecticide-treated bed nets in the area (Odhiambo et al. 2012). Surveillance continued after the completion of the trial, and Siaya was formalized as an HDSS site in 2001. The HDSS initially consisted of two communities (Asembo and Gem) and was expanded to a third (Karemo) in 2007. Malaria is endemic to the area, and HIV and tuberculosis prevalence are among the highest in Kenya (Hamel et al. 2011).

The cohort from Siaya consists of 28,877 births that took place between 2010 and 2014. Routine data collection rounds occurred three times per year up to 2014 and switched to twice per year thereafter. This transition did not affect the registration of pregnancies or births in the analytical cohort, and if it affected the reporting of mortality, it was not during the first year of life. At each household interview, fieldworkers collected information on pregnancy status and recent pregnancy outcomes from women of reproductive age (approximately 15–49). If a woman was not present at the time of interview, the questions were posed to a household proxy respondent. Reports of pregnancies were used to prompt fieldworkers to follow up on pregnancy outcomes. Prompts continued until an outcome was reported or the woman was lost to follow-up and the event was censored. In cases where the pregnancy was not reported, births were recorded without any pregnancy registration. A parallel continuous village reporter system was also used to collect information on births and deaths but not pregnancy reports.

#### Somkhele HDSS.

The Africa Health Research Institute (AHRI) conducts demographic surveillance activities from its Somkhele Research Campus in the uMkhanyakude District Municipality of KwaZulu-Natal province, South Africa (AHRI 2021). In 2000, surveillance was initiated in a study area of 438 km^2^ with a population of approximately 85,000 individuals (Gareta et al. 2021). In 2017, the size of the study area nearly doubled, and surveillance was expanded to a population of approximately 140,000 (Gareta et al. 2021). The area is predominantly rural, although it contains an urban township and some peri-urban settlements. HIV has severely affected the population since the start of the epidemic (Gareta et al. 2021). In 2003 HIV prevalence was 51 per cent for women aged 25–29 (Tanser et al. 2008).

This analysis is based on 4,633 births that took place in Somkhele HDSS between 2001 and 2004. Regular data collection rounds were conducted twice annually with the exception of 2002, when there were three rounds. At each household interview, basic data were collected from the household proxy respondent (typically the head of the household) or the next available senior adult household member. The proxy respondent reported the pregnancy statuses and recent pregnancy outcomes of female household members aged 15 years and older. Data collection forms were prepopulated with information collected in previous rounds. This would inform the interviewer if the individual in question had recently been pregnant, prompting them to inquire about the pregnancy outcome if one had not yet been reported.

## Methods

### Reporting of pregnancies and births

We began by identifying births that were registered as pregnancies at each site. This was straightforward for registered pregnancies that had been successfully followed up with pregnancy outcomes. However, it was not uncommon to observe stand-alone pregnancy registrations for which outcomes were never ascertained. When possible, we inferred the outcomes of such pregnancies from separate records of births or records of children residing in the HDSS who were linked to the mother. Pregnancy registrations were matched to outcomes occurring in the following 44 weeks. This window, which is four weeks longer than the length of an average pregnancy, was used to allow for matching in the presence of imprecision in date of birth reporting, which was common at each site. Pregnancy registrations from the day before or same day as a birth or within the same data collection round were discounted. We considered these to have been imputed and not reflective of prospective follow-up. We evaluated when each subject was first enumerated in the HDSS, whether prior to birth (in a pregnancy registration) or after birth (in a birth registration).

We continued with a descriptive analysis of child-, household-, and mother-level characteristics of births with and without pregnancy registrations at each site. A household wealth index was calculated separately for each site as a weighted average of variables denoting socio-economic status. Weights were generated from a principal component analysis, which included such variables as access to electricity, type of toilet facility, water supply source, and ownership of assets (e.g. radio, television, cooking stove). Information on HIV status was available for mothers in the South African and Kenyan sites. It was not available for Basse HDSS; however, this was not expected to be a limitation, given the extremely low prevalence of HIV in The Gambia (UNAIDS 2020). Mothers were coded as HIV-negative if they had received a negative test result any time after the birth of interest. Alternatively, they were labelled as either HIV-positive if they had received a positive test result prior to the delivery or within six months postpartum or as of unknown HIV status in all other cases. Mothers were considered recent migrants if they had resided outside the HDSS area for any part of the year preceding the delivery. Internal moves within the HDSS site were not considered migrations.

### Estimates of U5M

We calculated mortality schedules separately for pregnancy and birth cohorts. Deaths were tallied for the cohorts by single days of age from birth to five years. In the HDSS data, dates of events are recorded but not the ages at which they occur, so we deduced age at time of death as the difference between the event date and date of birth. Depending on the exact timing of these events, there is a margin of error of one day in age at death, which is inconsequential for deaths occurring after several months of life but significant for deaths within the first week. For example, a considerable number of deaths recorded as occurring the day after birth actually represent a lifespan of less than 24 hours (e.g. birth at 10 p.m., death at 3 a.m. the following day). To address this issue, we transformed the data from cohort–period format to age–cohort format by shifting half of all events to the previous day. Individual children contributed exposure time to the denominator of the mortality rate calculation while they were residing in the HDSS site. Given that deaths taking place outside the site would not have been captured by the HDSS, returning migrants did not add exposure time while they were living outside the surveillance area. Deaths and exposure time were aggregated across age groups to calculate abridged-life-table rates (_n_m_x_) for the following exact ages: 7, 14, 21, 28 days; 2, 3, 4, 5, 6, 7, 8, 9, 10, 11, 12, 15, 18, 21 months; and 2, 3, 4, 5 years (Guillot et al. 2022). Mortality rates for each cohort were converted into cumulative probabilities of dying (Q(x)) below age five under the assumption that mortality rates were constant within the age interval.

### Regression analysis

Aggregate differences in the mortality of the pregnancy and birth cohorts were investigated using micro-level analysis. We used inverse probability weighted (IP-weighted) Cox models to estimate the effect of pregnancy registration on U5M, controlling for potential selection bias from mothers or households that were more likely to register pregnancies. This approach was adapted from work on marginal structural models by Robins et al. (2000) and subsequent applications to survival analysis (Hernán et al. 2000; Buchanan et al. 2014). Inverse probability weights (IPWs) were used to standardize pregnancy and birth cohorts with respect to observable characteristics. Subjects in the pregnancy cohort were weighted by the inverse probability of pregnancy registration, while those in the birth cohort were weighted by the inverse probability of non-registration. In both cases, births that were extremely likely to have the expected pregnancy registration status (i.e. births in birth cohort that were likely to be lacking pregnancy registration based on observable characteristics and births in the pregnancy cohort that were likely to have an accompanying pregnancy registration) were weighted downwards, while unlikely members of either group were weighted upwards.

The IP-weighted Cox models were fitted by maximizing the weighted partial likelihood:

(1) $$L(\gamma ,\beta )=\underset{\delta_{i}=1}{\prod }\left[\frac{w_{i}\;\text{exp}\left(\gamma Z_{i}+X_{i}^{T}\beta \right)}{\underset{j\in R\left(t_{i}\right)}{\sum }w_{j}\;\text{exp}\left(\gamma Z_{j}+X_{j}^{T}\beta \right)}\right]$$

where *w*_*i*_ was the IPW for individual child *i*, *γ* was a coefficient for the binary indicator of pregnancy registration, *Z*; and *β* was a vector of coefficients for other covariates, *X*. The denominator was summed over all children still at risk at time *t*, and *δ*_*i*_ was an indicator for whether the observation ended in death or censoring (‘1’ or ‘0’, respectively).

The IPW for individual child *i* was defined as:

(2) $$w_{i}=\frac{P(Z)}{P\left(Z_{i}|X_{i}\right)}$$

where the covariate-conditional probability of pregnancy registration was estimated using probit regression. IPWs were stabilized by multiplying by the marginal probability of pregnancy registration in the numerator, as estimated in a separate probit model with no covariates (Robins et al. 2000). Decisions regarding which explanatory variables (*X*) to include in the weight model were made with the goal of balancing the pregnancy and birth cohorts with respect to covariates that were prognostically important (associated with U5M) or confounding (influencing both pregnancy registration and U5M) (Austin and Stuart 2015). In the former group, we included the sex of the child (UN IGME 2024), household wealth (Van Malderen et al. 2019), mother’s HIV status (Zaba et al. 2003), and whether the child had a sibling aged under 18 months at the time of birth (Bocquier et al. 2021). For potential confounders, we included the month of the child’s birth (Dorélien 2015), the mother’s age (Yaya et al. 2018), education level (Van Malderen et al. 2019), ethnicity (for Basse only), and marital status (Clark and Hamplová 2013; Yaya et al. 2018), and the household locality within the HDSS (Van Malderen et al. 2013). We excluded variables that were predictive of pregnancy registration but not U5M, as prior evidence indicates that such variables may result in increased bias and variance of the treatment effect in the main outcome model (Austin and Stuart 2015; Hernán and Robins 2020).

We calculated pseudo-*R*^2^ values to assess goodness of fit of the probit weight models (McFadden 1974). High goodness of fit (pseudo-*R*^2^ values of 0.2–0.4) (McFadden 1977) was considered indicative of a covariate imbalance between the pregnancy and birth cohorts and of a potential confounding effect on the relationship between pregnancy registration and U5M. We also calculated the overall accuracy of the models, defined as the proportion of individuals whose pregnancy registration status was correctly predicted using a probability cut-off of 50 per cent. For comparison, we fitted additional probit models that included covariates for whether a household interview took place in the 20 weeks prior to delivery and whether the mother self-reported at the interview (for Siaya and Somkhele HDSS only). These additional covariates, related to the timing and nature of data collected on pregnancy status, were considered unlikely to affect U5M (and thus inappropriate to include in the model used to generate IPWs) but likely associated with pregnancy registration.

We fitted four Cox models for each site: for neonatal (birth up to 28 days), postneonatal (28 days up to one year), child (one year up to five years), and under-five (birth up to five years) age groups. We fitted two unweighted models, regressing survival status on: (1) the binary indicator for pregnancy registration; and (2) the pregnancy registration indicator plus all covariates included in the probit weight model. We also fitted two IP-weighted models: (3) without adjustment for additional covariates; and (4) with such adjustment. The latter is similar to a doubly robust estimator: robust to misspecification in the weight model or Cox model but not both (Funk et al. 2011). We calculated standardized hazard ratios for pregnancy registration with robust standard errors. The proportional hazards assumption was verified graphically for the unweighted and IP-weighted samples. All statistical analysis was performed using R version 3.6.1.

## Results

### Reporting of pregnancies and births

**Figure 2:**
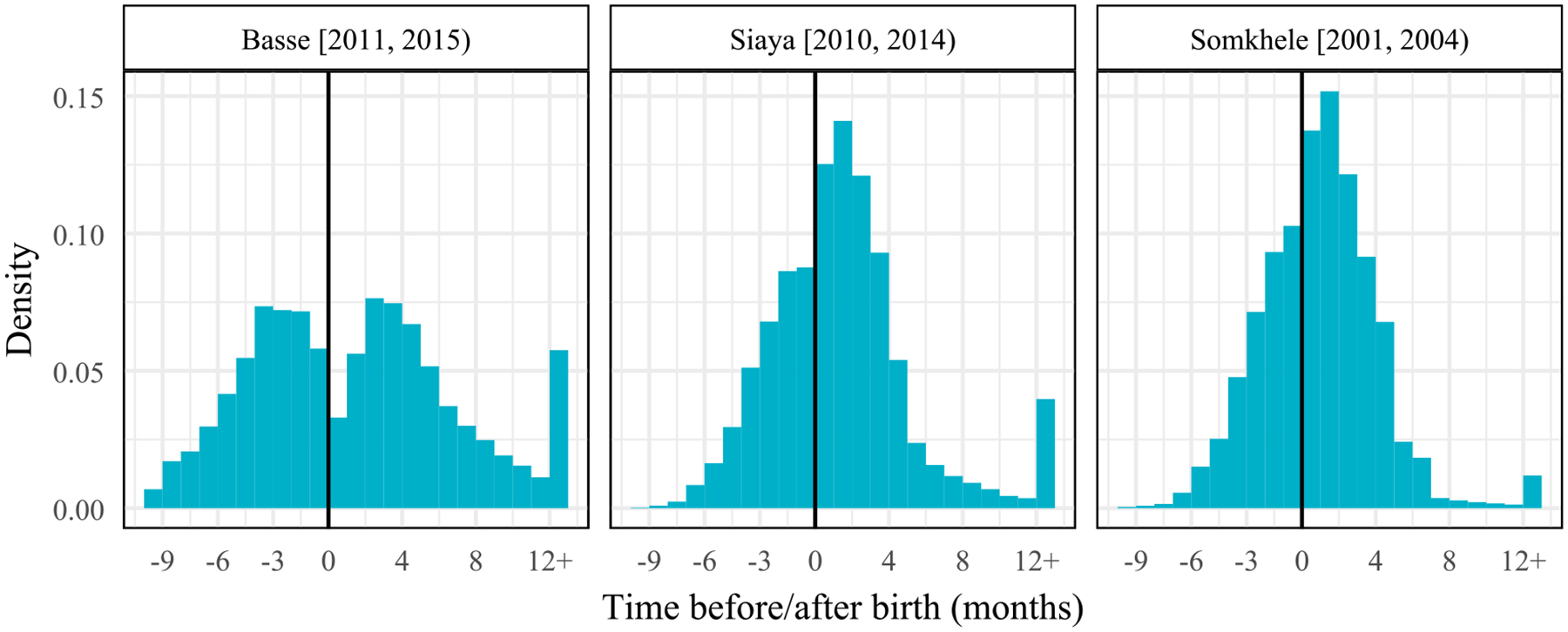
Relative distribution of when children were first enumerated (either as a pregnancy or birth) at three HDSS sites *Notes*: Births with pregnancy registration are plotted as negative values, showing when the pregnancy registration took place prior to the birth. For births lacking pregnancy registrations, the duration between birth and enumeration is shown. In Siaya and Somkhele HDSSs, the date of enumeration was approximated from the first household visit following a birth. *Source*: Authors’ analysis of data from HDSS sites in Basse [2011, 2015), Siaya [2010, 2014), and Somkhele [2001, 2004).

Figure 2 shows the date of first observation, whether as a pregnancy or birth, for all children included in the cohorts. Births with pregnancy registrations comprised 46 per cent of the total sample in Basse, 38 per cent in Siaya, and 41 per cent in Somkhele. Pregnancy registration was more likely to occur at later gestational ages, close to the date of delivery, at all sites. In Siaya and Somkhele HDSSs, the median pregnancy was registered around two to three months prior to birth, and around 80 per cent of pregnancies were registered in the four months prior to birth.

In Basse approximately 20 per cent of pregnancies were registered more than six months before birth, compared with 3–4 per cent at the other two sites. This reporting pattern is suspicious and likely includes imputed pregnancy registrations or reporting errors in dates of birth. Among observations lacking pregnancy registration in Basse, approximately 48 per cent were enumerated within four months of birth (i.e. during the first data collection round following birth).

For Siaya and Somkhele HDSSs, exact dates of enumeration were not available and were approximated from the first household interview following a child’s birth. As such, the distributions of enumerations for Siaya and Somkhele in Figure 2 represent the best-case scenario, in which all births were enumerated as soon as possible. Siaya HDSS was conducting triannual data collection rounds during this time, and for 78 per cent of births lacking a pregnancy registration, a household interview took place in the four months after the birth. For the remainder, it appears that there was irregularity in the timing of household visits or data-integrity issues regarding the harmonization of household visit records. In Somkhele HDSS, 95 per cent of births were linked with households that were visited in a data collection round within six months. This indicates that the site’s biannual rounds were comprehensive and consistent.

Table 1 summarizes child-, household-, and mother-level characteristics of the pregnancy and birth cohorts. In the Siaya HDSS pregnancy cohort, a household interview took place in the 20 weeks prior to delivery in 66 per cent of cases. Within the birth cohort, only 52 per cent of households were interviewed during the same period. The proportion with a household interview in the 20 weeks prior to delivery was also higher in the pregnancy cohort than the birth cohort in Somkhele. In the pregnancy cohort, 61 per cent of mothers were present at the time of the household interview, and 56 per cent self-reported their pregnancy status. Among those lacking pregnancy registration in Somkhele, only 25 per cent of mothers self-reported their pregnancy status at the interview taking place in the 20 weeks prior to delivery.

In Siaya, the Asembo and Gem localities reported slightly higher proportions of births with pregnancy registrations than Karemo. In Basse HDSS, the largest share of births without pregnancy registrations belonged to the bottom wealth quintile. In Somkhele, births with pregnancy registrations tended to belong to poorer households. There was no clear trend by household wealth quintile in Siaya. In Basse and Siaya HDSSs there were small but significant differences in the ages of pregnancy and birth cohort mothers, where the former were slightly older. In Siaya, 83 per cent of mothers in the pregnancy cohort had received no education or only primary education, compared with 79 per cent of mothers in the birth cohort. In Basse, mothers of Fula and Mandinka ethnicity made up a larger share of the pregnancy cohort than the birth cohort, whereas Sarahule women were more likely to be lacking pregnancy registration. In Siaya and Somkhele there was no statistically significant difference in the HIV status of mothers with and without pregnancy registration. At all sites, mothers in a formal union made up a larger share of the pregnancy cohort than the birth cohort. The difference was especially notable in Somkhele HDSS, where 95 per cent of mothers in the pregnancy cohort were in a union, compared with only 77 per cent of those in the birth cohort. In each site, pregnancy registration was less common for women who already had another child aged under 18 months. In Basse and Siaya, women who had recently migrated were less likely to have registered their pregnancies. There were no statistically significant differences between the pregnancy and birth cohorts in the sex ratio at birth at any site.

### Estimates of U5M

**Figure 3:**
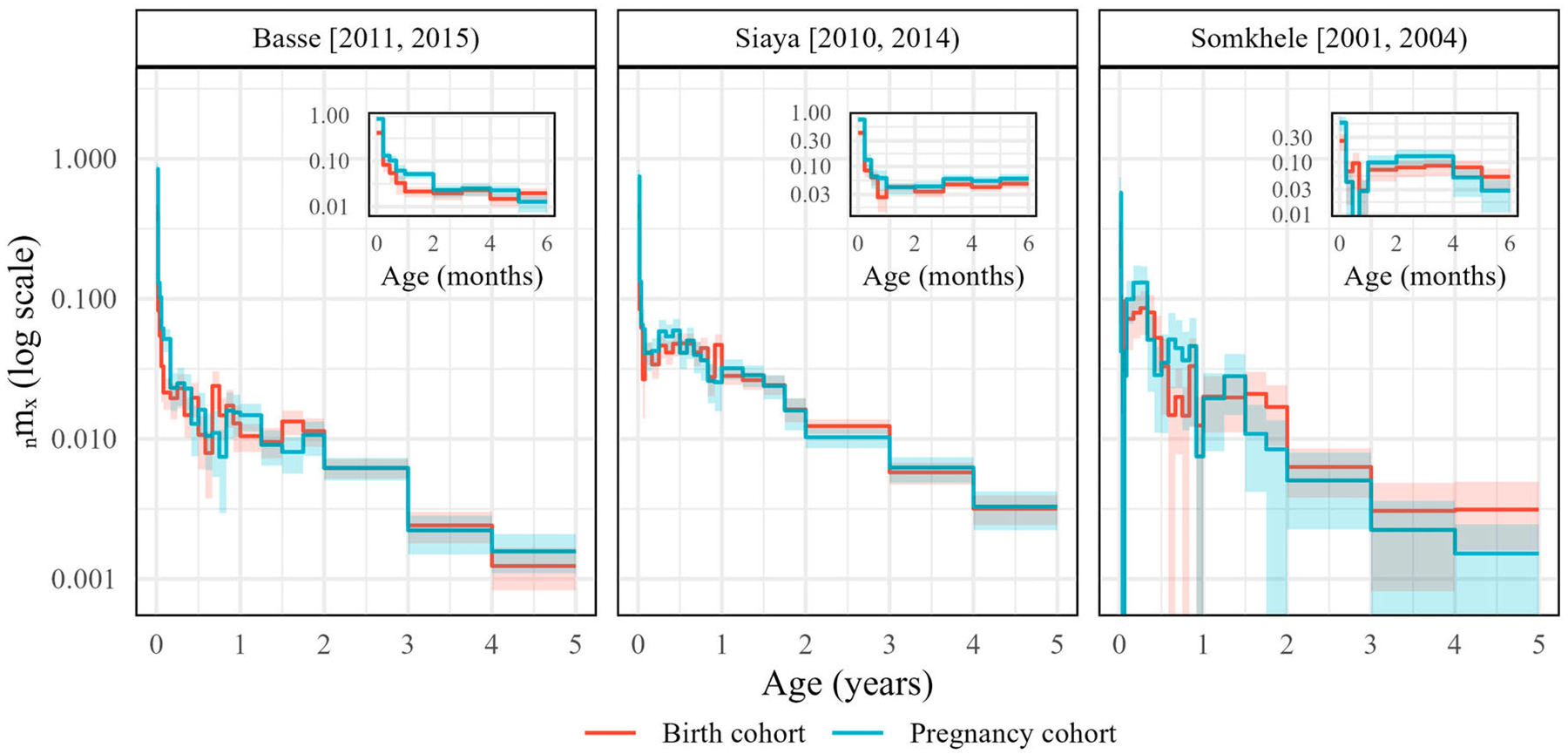
Age-specific mortality rates (_n_m_x_) for children under five years: Birth cohort vs pregnancy cohort at three HDSS sites *Notes*: Inset panels show mortality at ages below six months. Shaded areas show bootstrap 95 per cent confidence intervals, calculated for 5,000 resamples with replacement. *Source*: As for Figure 2.

Figure 3 displays the mortality schedules for the birth and pregnancy cohorts. During the first week of life, observed mortality for newborns in the pregnancy cohort was higher than in the birth cohort at all sites. In Basse HDSS, mortality rates in the pregnancy cohort were significantly higher for the first two months of life before becoming similar to those observed in the birth cohort. For Siaya HDSS, pregnancy cohort mortality rates were significantly higher in the first, second, and fourth weeks of life. There was also a slight increase in mortality risk between months three and six, which was more visible in the pregnancy cohort. In Somkhele HDSS, the difference between mortality rates for the pregnancy and birth cohorts was not significant after the first week of life. In the pregnancy cohort, the mortality rates for ages two months up to five months were higher than those of the late neonatal period (ages two to four weeks), perhaps indicating issues related to small sample size and data quality. Across all sites, mortality rates for the pregnancy and birth cohorts became more similar at older ages.

**Figure 4:**
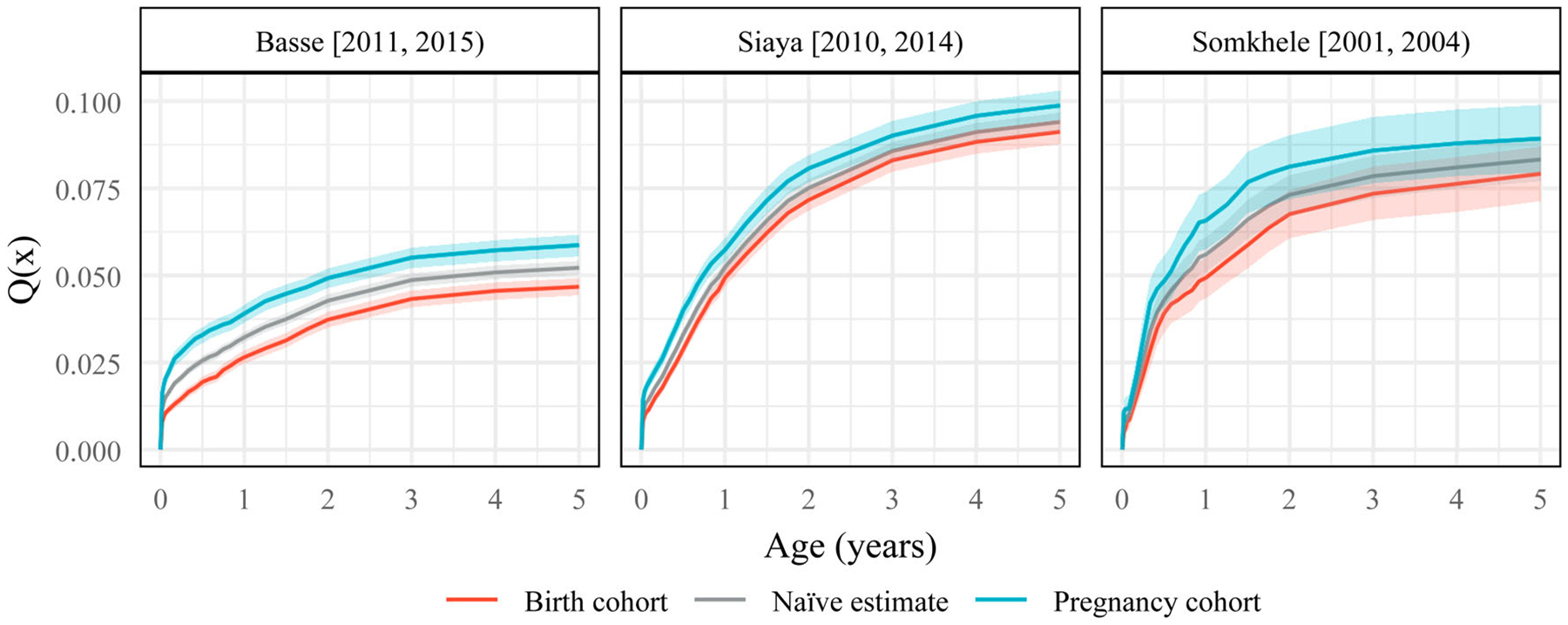
Cumulative probabilities of dying (Q(x)) by age five years: Birth cohort, pregnancy cohort, and naïve estimate for three HDSS sites *Note*: Shaded areas show bootstrap 95 per cent confidence intervals, calculated for 5,000 resamples with replacement. The naïve estimate was calculated from all births combined. *Source*: As for Figure 2.

The cumulative probabilities of dying below age five for each cohort are shown in Figure 4. This figure also includes a naïve mortality estimate calculated from all births combined. The pregnancy cohort yielded the highest estimate of U5M for each site. In Basse, the cumulative probability of dying by age five for children in the birth cohort was 47 per 1,000 births (95 per cent confidence interval [CI] 0.044–0.049). In the pregnancy cohort, it was 12 deaths per 1,000 births higher, at 59 per 1,000 (95 per cent CI 0.056–0.062). The naïve estimate for all births was between these two values, at 52 per 1,000 (95 per cent CI 0.050–0.054).

In Siaya HDSS, the cumulative probability of dying by age five was close to 10 per cent in the pregnancy cohort (0.099, 95 per cent CI 0.095–0.103). Observed mortality in the birth cohort was 92 per 1,000 (95 per cent CI 0.088–0.095), and the naïve estimate was 94 per 1,000 (95 per cent CI 0.092–0.097). In Somkhele HDSS, mortality in the pregnancy cohort was 89 per 1,000 (95 per cent CI 0.079–0.100). This was five deaths per 1,000 higher than the naïve estimate, and 10 deaths per 1,000 births higher than the estimate for the birth cohort. However, the difference between the mortality of the pregnancy and birth cohorts by age five was not as statistically significant as for the other two sites and the confidence intervals overlapped.

### Regression analysis

Table 2 displays the results of the probit models regressing pregnancy registration on observable characteristics of the child, household, and mother. The table reports the estimated marginal effect of each covariate on the probability of pregnancy registration. At each site, mothers who already had a young child were 10–17 per cent less likely to register their pregnancy. Household locality was important in Siaya, where mothers residing in Asembo were roughly 15 per cent more likely to have registered their pregnancy compared with mothers in Gem or Karemo. The coefficients for household wealth were most significant in Siaya, where those in the highest wealth quintile were least likely to have registered their pregnancy. Month of birth was a significant covariate at each site, but the marginal effects were largest for Somkhele.

While the covariate for mother’s age was significantly associated with pregnancy registration at each site, the marginal effect of this variable was very small. In Siaya and Somkhele, being of unknown HIV status was associated with a reduced probability of pregnancy registration, perhaps indicating that such individuals were less likely in general to have complete information recorded in the HDSS. Marital status had the strongest effect on pregnancy registration of all variables in Somkhele: unpartnered women and women of unknown marital status were 30 and 45 per cent, respectively, less likely to have registered their pregnancy than women in a union. The same relationship was found for the other sites but with smaller effects. Being a recent migrant significantly decreased the probability of pregnancy registration in Basse and Siaya.

Despite the presence of several significant covariates, goodness of fit was somewhat low by traditional standards for binary classification models. The lowest pseudo-*R*^2^ value, 0.04, was found in the model for Basse HDSS, while values for Siaya and Somkhele HDSSs were slightly higher, at 0.06 and 0.08, respectively. The model for Basse correctly predicted pregnancy registration in 59 per cent of cases. This was only slightly higher than the no-information rate (NIR) of 54 per cent. There was a difference of two percentage points between accuracy (0.64) and NIR (0.62) for the Siaya model and of four percentage points for Somkhele (accuracy = 0.63, NIR = 0.59). Thus, predicting pregnancy registration at the individual level was difficult, and there was substantial randomness in pregnancy registration that was not well explained by the covariates in the model.

For Siaya and Somkhele HDSSs, we fitted additional probit models which included more variables related to data collection (see Appendix Table A1). The model for Siaya included an indicator for whether a household interview took place less than 20 weeks prior to delivery. This model showed slightly higher goodness of fit (pseudo-*R*^2^ = 0.07) and predictive accuracy (accuracy = 0.66) than the model in Table 2. For Somkhele, the household interview covariate also included information for whether the mother self-reported her pregnancy status. This resulted in a larger improvement in predictive performance (pseudo-*R*^2^ = 0.13, accuracy = 0.69). Women who self-reported their pregnancy status at a household interview taking place less than 20 weeks prior to delivery were 27 per cent more likely to have registered their pregnancy.

**Figure A1:**
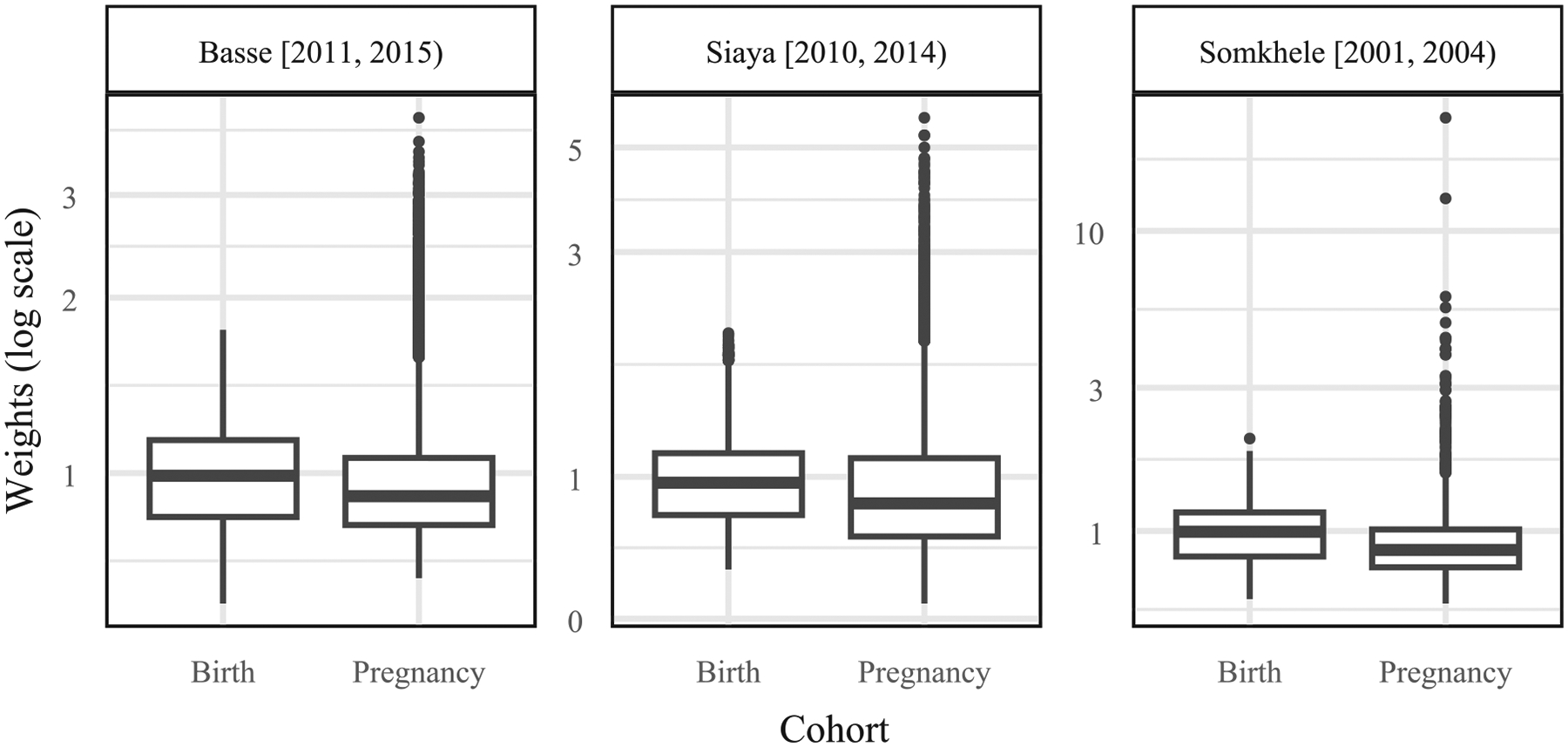
Distribution of inverse probability of pregnancy registration weights for birth and pregnancy cohorts at three HDSS sites *Source*: Authors’ analysis of data from HDSS sites in Basse [2011, 2015), Siaya [2010, 2014), and Somkhele [2001, 2004).

The issue of selectivity in pregnancy registration was investigated further using Cox regression. The predicted probabilities of pregnancy registration from the probit models were transformed into IPWs with mean values close to 1.0 for pregnancy and birth cohorts (see Appendix Figure A1).

**Figure 5:**
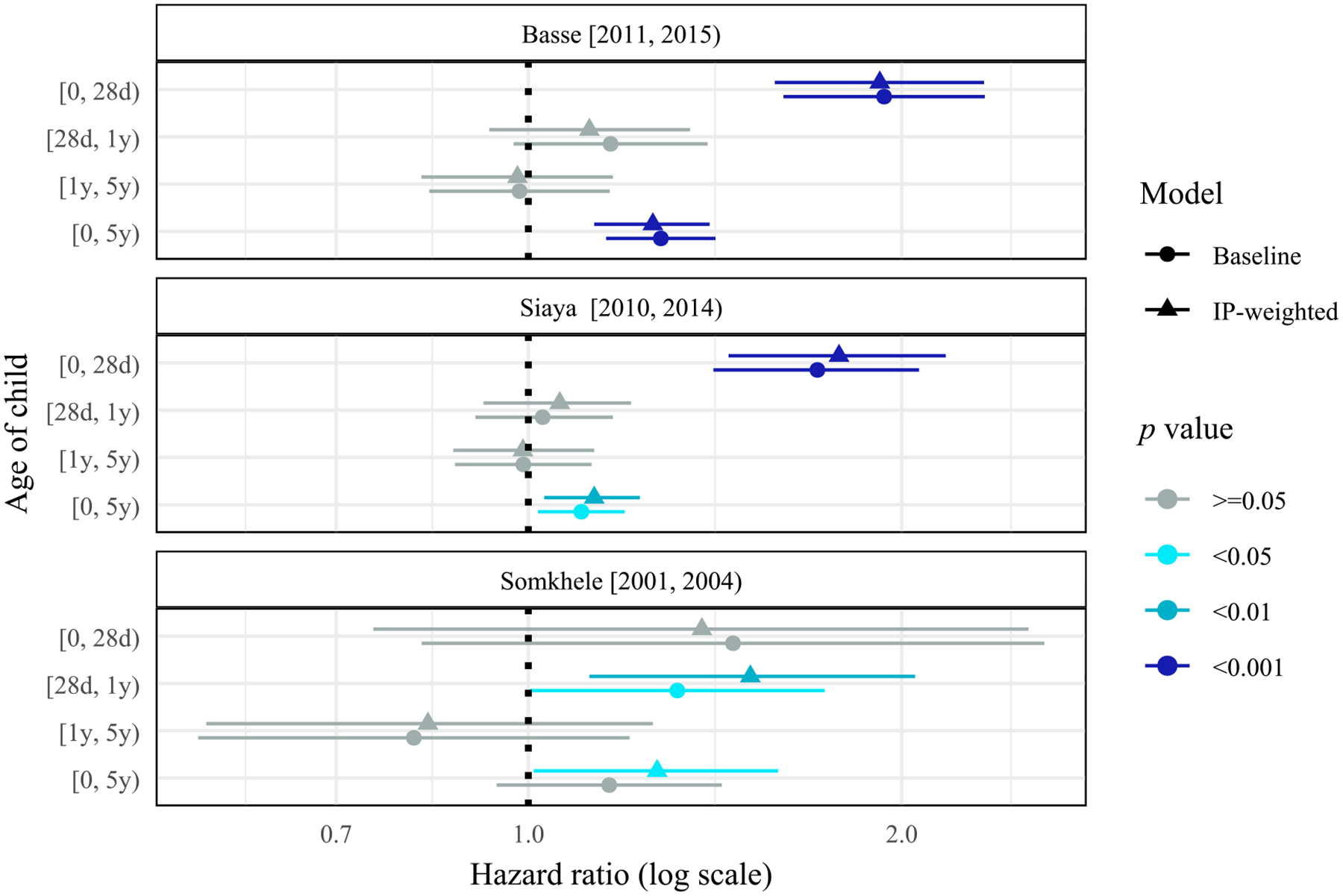
Results from Cox regression models for mortality under age five (pregnancy cohort vs birth cohort) at three HDSS sites *Notes*: Estimated hazard ratios for births with a prior pregnancy registration (relative to those without a pregnancy registration) are displayed as dots with horizontal lines for 95 per cent confidence intervals for the unweighted baseline model (Model 1) and IP-weighted model with no covariates (Model 3). Inverse probability weighted (IP-weighted) estimates are shown with robust 95 per cent confidence intervals. *Source*: As for Figure 2.

Results for two of the Cox models regressing child survival on pregnancy registration are shown in Figure 5. The figure displays the hazard ratios for pregnancy registration in the unweighted baseline model (Model 1) and the IP-weighted model with no covariates (Model 3). In the unweighted baseline model for Basse HDSS, newborns in the pregnancy cohort displayed almost double the observed risk of neonatal mortality (hazard ratio [HR] 1.94; 95 per cent CI 1.61–2.33) compared with the birth cohort. After standardizing for covariate imbalances between the pregnancy and birth cohorts using the IPWs, the hazard ratio decreased slightly (HR 1.92; robust 95 per cent CI 1.58–2.33). This indicated that the relationship between pregnancy registration and neonatal mortality was subject to some positive confounding in Basse HDSS. Adjustment from IPWs also reduced the hazard ratio for pregnancy registration in the U5M model from 1.28 (95 per cent CI 1.16–1.42) to 1.26 (robust 95 per cent CI 1.13–1.40).

As in Basse, the observed risk of dying in the neonatal period in Siaya HDSS was significantly higher for newborns in the pregnancy cohort than for the birth cohort. In the baseline model, newborns with pregnancy registrations were 1.71 (95 per cent CI 1.41–2.07) times more likely to die at neonatal ages compared with those without. However, unlike Basse, adjustment with IPWs led to an increased hazard ratio in Siaya of 1.78 (robust 95 per cent CI 1.45–2.17). This suggested that pregnancy registration in Siaya was associated with characteristics that were protective of mortality. As such, after controlling for characteristics associated with pregnancy registration and mortality using the IPWs, the hazard ratio for pregnancy registration increased even further.

For total U5M, the risk in the pregnancy cohort was also slightly higher than in the birth cohort in Siaya. In the baseline model, children with registered pregnancies experienced 1.10 times the risk of U5M (95 per cent CI, 1.02–1.20). The hazard ratio increased and became more significant in the model adjusted with IPWs (HR 1.13; robust 95 per cent CI, 1.03–1.23). In neither Basse nor Siaya was the hazard ratio for pregnancy registration highly significant in the postneonatal or child age groups.

Somkhele differed from Basse and Siaya in displaying important differences between the observed postneonatal mortality of the pregnancy and birth cohorts. Compared with the birth cohort, the pregnancy cohort showed an approximately 51 per cent higher risk of postneonatal mortality in the IP-weighted model (HR 1.51; robust 95 per cent CI 1.12–2.05). This was an increase from the baseline estimate of 1.32 (95 per cent CI 1.00–1.73). In the baseline models for U5M, mortality risk was not significantly different between pregnancy and birth cohorts. After adjustment with IPWs, the pregnancy cohort showed a 27 per cent higher risk for U5M, with moderate significance (robust 95 per cent CI 1.01–1.59).

For all sites, the hazard ratios of IP-weighted models were similar to those of the other Cox models included in the sensitivity analysis (i.e. unweighted, covariate-adjusted models and double robust models adjusted with both weights and covariates). Full results are provided in Appendix Table A2.

## Discussion

Using data from HDSS sites in Basse (The Gambia), Siaya (Kenya), and Somkhele (South Africa), we found that estimates of U5M for children whose births were preceded by a pregnancy registration were higher than for those lacking a pregnancy registration. This difference in observed mortality was attributable primarily to mortality in the neonatal and postneonatal periods. In Basse and Siaya HDSSs, the pregnancy cohort experienced higher mortality than the birth cohort in the first few weeks of life. For Somkhele HDSS, the mortality of the pregnancy cohort was significantly higher during the first week of life. Overall, pregnancy registration seemed to result in improved or increased ascertainment of early deaths. For all sites, mortality of the pregnancy and birth cohorts was not statistically different between the ages of one and five years.

The proportion of births with a pregnancy registration comprised a minority of each sample and was relatively similar across sites (38–46 per cent). Given that Basse and Siaya HDSSs were conducting triannual data collection rounds compared with Somkhele’s biannual rounds, it was somewhat surprising that these sites did not report substantially higher pregnancy registration. Indeed, if births were randomly distributed throughout the year, and under the conservative assumption that pregnancy registration would occur only if a data collection round took place during the third trimester, triannual interviews would theoretically result in around 75 per cent of pregnancies being registered. That this level was not achieved at either site indicates considerable under-reporting. Furthermore, it suggests that marginally increasing the frequency of interview rounds alone would not be sufficient to achieve exhaustive pregnancy registration.

At all sites, the frequency of pregnancy registration generally increased with gestational age. However, it was also not uncommon for a household interview to have occurred during the later stages of pregnancy and for there to be no pregnancy registration. A household interview took place in the 20 weeks prior to delivery for 52 per cent of those in the birth cohort in Siaya and 76 per cent in Somkhele. The lack of pregnancy registrations in such cases could be related to the fact that women often travel to seek medical care or family support around the time of birth in these settings (Clouse et al. 2018). Our results suggest that factors related to proxy reporting also play an important role in pregnancy registration. In Somkhele HDSS, a pregnancy was more likely to be registered if the woman was present during the household visit of the HDSS team and self-reported her own pregnancy status. Across all three sites, women in a union were more likely to have registered their pregnancy. Under-reporting of pregnancies for women not in a formal union could be related to strong social norms against having a child outside marriage. It is also the case that married women often reside in households where the household head (and likely proxy respondent) is their husband. While women in rural sub-Saharan Africa often conceal their pregnancy from the broader community for as long as possible, they typically disclose their pregnancy status to a sexual partner soon after the first missed menses, to secure support in preparing for the pregnancy (Haws et al. 2010). Thus, proxy reporting likely contributes to the under-reporting of all pregnancies but especially those of women who are living with their partner.

Given the incompleteness of pregnancy registration in the HDSS, it was important to examine whether the higher mortality of pregnancy cohorts was due to these observations being negatively selected in terms of U5M risk factors. We used Cox regression models with IP weighting to control for selection bias and estimate the marginal effect of pregnancy registration on mortality below age five. For constructing the IPWs, probit models were used to estimate the effect of child-, household-, and mother-level characteristics on the probability of pregnancy registration. These models showed low goodness of fit for each site, indicating that pregnancy registration was poorly predicted by observable characteristics. This finding indicated that pregnancy and birth cohorts did not differ substantially in terms of measurable risk factors for U5M, thus suggesting low potential for a confounding effect between pregnancy registration and child survival. We sought to confirm this through survival analysis.

Observed mortality in the pregnancy cohort remained higher after standardizing pregnancy and birth cohorts for covariate composition using the IPWs. In the case of Siaya and Somkhele HDSSs, the hazard ratios increased or became more significant. In Basse, there was a small reduction in the baseline effect of pregnancy registration on U5M after adjustment, although it remained significant. These results support the proposition that mortality estimates for pregnancy cohorts are an improvement over naïve estimates and one that is not attributable to selection bias.

There have long been concerns regarding the quality of early mortality data in HDSSs. Systematic comparison with corresponding subnational estimates from cross-sectional surveys such as MICS and DHS have shown HDSSs to capture lower levels of neonatal mortality and higher levels of mortality at postneonatal and child ages (Eilerts et al. 2021). While retrospective surveys may perform better in estimating early mortality, tracking mortality for older children who have already been enumerated plays to the strengths of prospective surveillance in HDSSs. Our findings suggest that HDSS pregnancy cohort estimates represent the best of both worlds in terms of correcting for downward bias in HDSS estimates of early mortality and harnessing the robust surveillance for older child ages. As such, these estimates can also be leveraged to improve understanding regarding the age pattern of U5M in African settings and to disentangle whether deviation from established age patterns is due to a unique epidemiological environment or data-quality issues.

This study has drawn on the richness of HDSS data to investigate macro-level differences in the mortality of cohorts with and without pregnancy registrations, controlling for micro-level factors. However, the study was subject to some important weaknesses. Pregnancy registration was not randomly assigned in a controlled trial, and determining its effect on measurement of U5M was not straightforward. While the IPWs used in the Cox regression models controlled confounding by measured covariates, they did not adjust for potential bias from unmeasured variables. There was thus the possibility for residual confounding of the association between pregnancy registration and U5M by omitted or insufficiently controlled for factors. Clinical markers are usually a better predictor of neonatal mortality and were not available for inclusion. However, for these to explain the higher estimates of mortality in pregnancy cohorts, they would also need to be strongly associated with pregnancy registration. There is no strong a priori reason to presume that this would be the case, especially in light of our finding that pregnancy registration was better predicted by the timing of data collection and use/non-use of a proxy respondent than by household- or mother-level characteristics.

**Figure A2:**
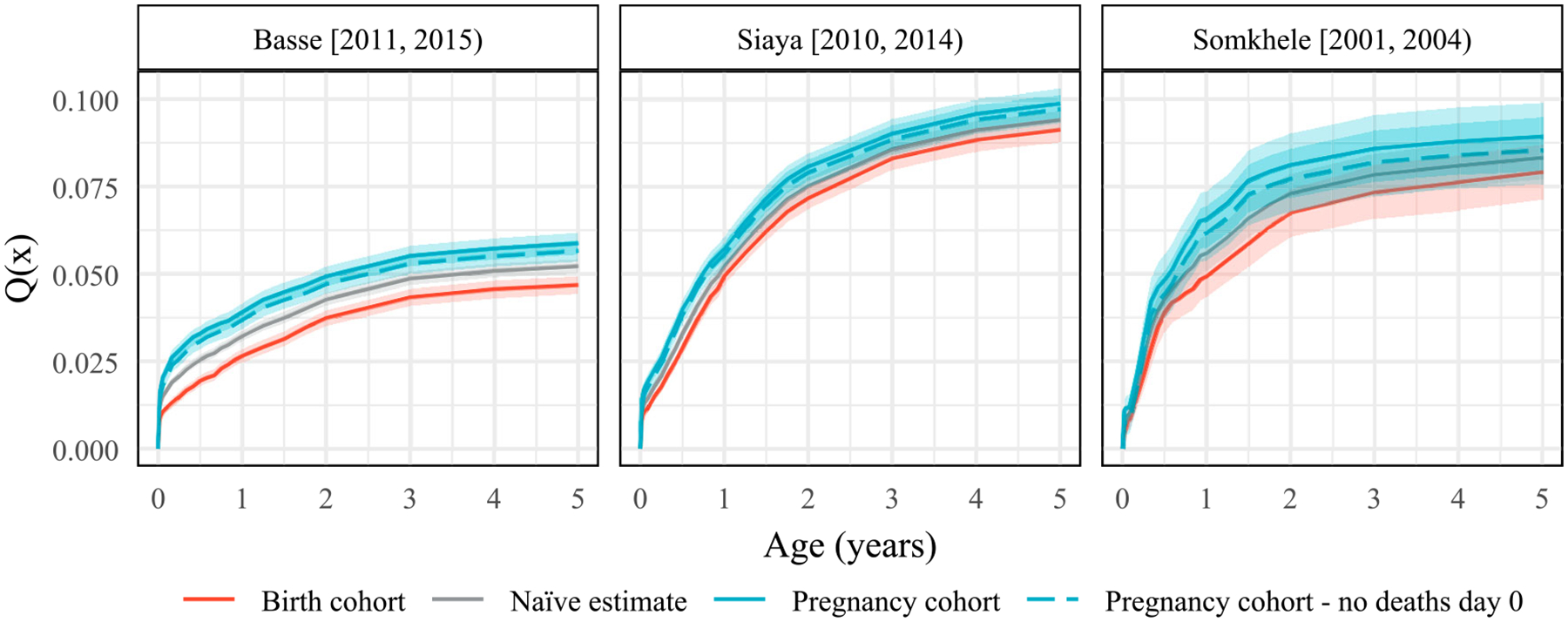
Sensitivity analysis: Cumulative probabilities of dying (Q(x)) by age five years assuming all deaths on the first day of life were stillbirths wrongly classified as neonatal deaths *Notes*: All deaths taking place during the first day of life were removed from the pregnancy cohort, and cumulative probabilities of dying under five were recalculated. Shaded areas show bootstrap 95 per cent confidence intervals, calculated for 5,000 resamples with replacement. *Source*: As for Figure A1.

Additionally, we did not investigate whether issues of misclassification of stillbirths and neonatal deaths differentially impacted pregnancy cohorts. Recent research has found evidence of potential overestimation of neonatal mortality in African DHS due to stillbirths being misreported as neonatal deaths (Liu et al. 2016; Helleringer et al. 2020). Such work raises important questions regarding the accuracy of data collected on early deaths in maternity history questionnaires; however, there has not been much investigation of issues of misclassification in HDSS data. Nevertheless, it has been observed that stillbirths that are misclassified as neonatal deaths are most likely to be recorded as very early deaths, taking place in the first or second day of life (Helleringer et al. 2020). To account for this, we conducted a sensitivity analysis where deaths taking place on the first day of life were omitted from the pregnancy cohorts. These results showed that even in the extreme case that all deaths taking place on the first day of life were stillbirths wrongly classified as neonatal deaths, the pregnancy cohort estimates of neonatal and under-five mortality would still be higher than birth cohort estimates for all sites (Appendix Figure A2). It is thus not likely that misclassification of stillbirths could fully account for the higher observed mortality in the pregnancy cohort group.

## Conclusion

Our research indicates that pregnancy registration improves follow-up on the vital events of newborns in HDSSs. This is not to say that early mortality estimates from pregnancy cohorts are entirely unaffected by omissions of early deaths; however, they are likely subject to less downward bias than estimates calculated from all births (irrespective of pregnancy registration status). Enhancing the quality and consistency of pregnancy data can address long-standing concerns surrounding the accuracy of HDSS estimates of early mortality so as to constitute a true gold standard for U5M data in settings lacking CRVS. Pregnancy registration can also contribute to the tracking of maternal health and pregnancy outcomes (e.g. stillbirths) that have not been systematically measured in high-burden countries. These potential pay-offs suggest that improving the quality and completeness of pregnancy data should be a top priority in HDSSs. In the context of the discontinuation of DHS surveys, the role of HDSSs as a critical source of information in these areas has become more important than ever.

As a first step, we recommend that HDSS protocols and procedures for collecting data on pregnancies be extensively documented. These details are often missing from site descriptions or publicly available data resource profiles, despite providing necessary context for the mortality and fertility estimates arising from such data. In terms of changes to data collection itself, conducting more frequent interview rounds is an appealing prospect although not always financially feasible. Additionally, while the frequency of data collection rounds is an important determinant of pregnancy reporting completeness, it is not the only determinant. Data collection procedures and protocols exert an enormous influence on pregnancy registration. HDSSs should strive to pose questions about pregnancy status to women directly and avoid proxy reports. At a minimum, the identity of the respondent should be recorded. At sites where direct reports are collected, further gains could be achieved by using female interviewers and training interviewers to collect data in a sensitive manner. Record linkage with routine programme data is another promising avenue for enhancing HDSS reporting. In areas where coverage of antenatal care services is high, sites could leverage such data to ascertain pregnancy-status information for residents. Of course, these efforts will all incur financial and logistical challenges of their own, and the implications for different methods of pregnancy surveillance on data quality and completeness must be considered. However, given the many benefits that pregnancy registration provides for monitoring maternal and newborn health in HDSSs, we argue that these steps would be a worthwhile investment. Such data will be essential for guiding evidence-based public health interventions and accelerating progress towards mortality reduction.

## Figures and Tables

**Table 1:** Characteristics of the pregnancy and birth cohorts at the Basse, Siaya, and Somkhele HDSS sites

	Basse HDSS [2011, 2015)			Siaya HDSS [2010, 2014)			Somkhele HDSS [2001, 2004)		
	*Pregnancy cohort*	*Birth cohort*	*Sig.*	*Pregnancy cohort*	*Birth cohort*	*Sig.*	*Pregnancy cohort*	*Birth cohort*	*Sig.*
*Household interview <20 weeks prior to delivery*	–	–		0.66	0.52	<0.01	0.89	0.76	<0.01
*Mother is present*									
No	–	–		–	–		0.28	0.47	<0.01
Yes	–	–		–	–		0.61	0.29	
Not applicable	–	–		–	–		0.11	0.24	
*Mother is self-respondent*									
No	–	–		–	–		0.33	0.51	<0.01
Yes	–	–		–	–		0.56	0.25	
Not applicable	–	–		–	–		0.11	0.24	
*Household locality*									
Asembo	–	–		0.34	0.25	<0.01	–	–	
Gem	–	–		0.35	0.36		–	–	
Karemo	–	–		0.31	0.39		–	–	
*Household wealth quintile*									
1	0.16	0.18	<0.01	0.15	0.14	<0.01	0.20	0.19	<0.01
2	0.16	0.16		0.16	0.15		0.21	0.18	
3	0.10	0.10		0.15	0.15		0.19	0.19	
4	0.17	0.14		0.17	0.16		0.18	0.18	
5	0.14	0.13		0.12	0.15		0.18	0.21	
Unknown	0.26	0.29		0.24	0.25		0.04	0.05	
*Mother’s age*	27.30	26.12	<0.01	25.97	25.19	<0.01	25.34	25.51	0.42
*Mother’s education level*									
None/primary	0.01	0.02	<0.01	0.83	0.79	<0.01	0.32	0.29	0.20
Secondary/religious	0.30	0.31		0.16	0.20		0.67	0.67	
Unknown	0.68	0.67		0.00	0.01		0.02	0.03	
*Mother’s ethnicity*									
Fula	0.34	0.31	<0.01	–	–		–	–	
Mandinka	0.23	0.20		–	–		–	–	
Sarahule	0.41	0.46		–	–		–	–	
Other	0.02	0.02		–	–		–	–	
*Mother’s HIV status*									
Negative	–	–		0.27	0.25	0.15	0.47	0.41	0.84
Positive	–	–		0.04	0.03		0.01	0.01	
Unknown	–	–		0.69	0.72		0.51	0.57	
*Mother’s marital status*									
In union	0.52	0.38	<0.01	0.79	0.64	<0.01	0.95	0.77	<0.01
Not in union^1^	0.48	0.62		0.15	0.25		0.05	0.16	
Unknown				0.06	0.11		0.00	0.07	
*Mother has child <18 months old*	0.02	0.03	<0.01	0.04	0.05	<0.01	0.02	0.04	<0.01
*Mother is recent migrant*	0.06	0.16	<0.01	0.14	0.28	<0.01	0.26	0.25	0.90
*Sex ratio at birth*	1.00	1.04	0.19	1.00	1.01	0.69	0.99	1.05	0.30
*N*	0.46	0.54		0.38	0.62		0.41	0.59	

1 In Basse HDSS, marital status was recorded only for women in a union. There was no information to distinguish those not in a union from those of unknown status.

*Notes:* Categorical variables are presented as weighted proportions and continuous variables as means. All measures are categorical except Mother’s age (ranges from 10 to 49) and Sex ratio at birth. Sex of child is displayed as the sex ratio here, but for the probability weighting in the analysis, it is taken into account at the individual level. Sig. refers to significance: two-sample *t* tests were performed for continuous outcomes, and chi-square tests were performed for categorical outcomes. Tests were performed on complete case data, with Unknown values excluded.

*Source*: Authors’ analysis of data from HDSS sites in Basse [2011, 2015), Siaya [2010, 2014), and Somkhele [2001, 2004).

**Table 2:** Results from probit regression analysis of pregnancy registration at the Basse, Siaya, and Somkhele HDSS sites

Outcome: Pregnancy registration	Basse [2011, 2015)	Siaya [2010, 2014)	Somkhele [2001, 2004)
*Child sex (ref. = Female)*			
Male	−0.01	0.00	−0.02
*Has child <18 months old (ref. = No)*			
Yes	−0.17***	−0.10***	−0.16***
*Household locality (ref. = Karemo)*			
Asembo	–	0.15***	–
Gem	–	0.01	–
*Household wealth quintile (ref. = 1)*			
2	0.01	0.00	0.02
3	0.01	−0.02^†^	−0.01
4	0.03**	−0.01	−0.01
5	0.01	−0.04***	−0.01
Unknown	−0.01	−0.03**	−0.01
*Month of birth (ref. = 1)*			
2	−0.01	−0.03*	0.01
3	0.02^†^	0.06***	0.10**
4	0.02	0.09***	0.14***
5	0.03^†^	0.04**	0.11***
6	−0.02	0.02	0.09**
7	0.00	0.03*	0.11***
8	0.00	0.08***	0.10**
9	0.04**	0.05***	0.19***
10	0.07***	0.03*	0.12***
11	0.05***	0.05***	0.07^†^
12	0.07***	0.08***	0.04
*Mother’s age*	0.00***	0.00***	−0.01***
*Mother’s education (ref. = None/Primary)*			
Secondary/religious	0.07**	−0.04***	−0.03^†^
Unknown	0.09***	−0.15***	−0.02
*Mother’s ethnicity (ref. = Sarahule)*			
Fula	0.05***	–	–
Mandinka	0.06***	–	–
Other	0.00	–	–
*Mother’s HIV status (ref. = Negative)*			
Positive	–	0.02	−0.07
Unknown	–	−0.03***	−0.05***
*Mother’s marital status (ref. = In a union)*			
Not in union^1^	–	−0.17***	−0.30***
Unknown	−0.09***	−0.19***	−0.45***
*Mother recently migrated (ref. = No)*			
Yes	−0.22***	−0.17***	0.03*
Observations	29,447	28,877	4,633
No-information rate	0.54	0.62	0.59
Accuracy	0.59	0.64	0.63
Pseudo R-squared	0.04	0.06	0.08

† *p* < 0.10;

* *p* < 0.05;

** *p* < 0.01;

*** *p* < 0.001.

1 In Basse HDSS, marital status was recorded only for women in a union. There was no information to distinguish those not in a union from those of unknown status.

*Note*: Table shows marginal effects.

*Source*: As for Table 1.

## Appendix

**Table A1:** Results from probit regression analysis incorporating additional covariates related to the timing of household interview, presence of the mother, and identity of the respondent

	Siaya [2010, 2014)	Somkhele [2001, 2004)
*Child sex (ref. = Female)*		
Male	−0.00	−0.01
*Has child <18 months old (ref. = No)*		
Yes	−0.10***	−0.17***
*Household interview <20 weeks prior to delivery (ref. = No)*		
Yes	0.14***	–
Yes, Self-respondent	–	0.27***
Yes, Proxy respondent	–	0.00
*Household locality (ref. = Karemo)*		
Asembo	0.16***	–
Gem	0.02**	–
*Household wealth quintile (ref. = 1)*		
2	−0.00	0.02
3	−0.01	−0.01
4	−0.00	−0.01
5	−0.05***	−0.01
Unknown	−0.04***	−0.02
*Month of birth (ref. = 1)*		
2	−0.03^†^	0.03
3	0.06***	0.09**
4	0.08***	0.13***
5	0.03*	0.09**
6	0.01	0.06^†^
7	0.02^†^	0.09**
8	0.07***	0.10**
9	0.04***	0.16***
10	0.03*	0.10**
11	0.04***	0.05
12	0.07***	0.05
*Mother’s age*	−0.00***	−0.01***
*Mother’s education (ref. = None/Primary)*		
Secondary/Religious	−0.04***	−0.02
Unknown	−0.14***	−0.02
*Mother’s HIV status (ref. = Negative)*		
Positive	0.02	−0.06
Unknown	−0.04***	−0.04**
*Mother’s marital status (ref. = In union)*		
Not in union	−0.17***	−0.26***
Unknown	−0.19***	−0.43***
*Mother recently migrated (ref. = No)*		
Yes	−0.17***	0.03*
Observations	28,877	4,633
No-information rate	0.62	0.59
Accuracy	0.66	0.69
Pseudo R-squared	0.07	0.13

† *p* < 0.10;

* *p* < 0.05;

** *p* < 0.01;

*** *p* < 0.001.

*Note*: Table shows marginal effects.

*Source*: Authors’ analysis of data from HDSS sites in Siaya [2010, 2014) and Somkhele [2001, 2004).

**Table A2:** Results from Cox regression analysis: Hazard ratios and 95 per cent confidence intervals for the effect of pregnancy registration on under-five mortality at three HDSS sites

HDSS	Age group	Pregnancy cohort		Birth cohort		(1) Unweighted HR (95 per cent CI)	(2) Unweighted HR (95 per cent CI) adjusted for covariates	(3) IP-weighted HR (robust 95 per cent CI)	(4) IP-weighted HR (robust 95 per cent CI) adjusted for covariates
		Deaths	Person-years	Deaths	Person-years				
Basse	[0, 28d)	288	1,012.09	178	1,214.42	1.94*** (1.61, 2.33)	1.90*** (1.57, 2.30)	1.92*** (1.58, 2.33)	1.92*** (1.58, 2.33)
	[28d, 1y)	233	11,813.77	241	14,231.39	1.16 (0.97, 1.39)	1.12 (0.93, 1.34)	1.12 (0.93, 1.35)	1.13 (0.93, 1.36)
	[1y, 5y)	251	47,847.24	302	56,268.24	0.98 (0.83, 1.16)	0.97 (0.82, 1.15)	0.98 (0.82, 1.17)	0.98 (0.82, 1.16)
	[0, 5y)	772	60,673.10	721	71,714.03	1.28*** (1.16, 1.42)	1.25*** (1.13, 1.39)	1.26*** (1.13, 1.40)	1.26*** (1.13, 1.40)
Siaya	[0, 28d)	210	827.49	201	1,355.79	1.71*** (1.41, 2.07)	1.76*** (1.44, 2.15)	1.78*** (1.45, 2.17)	1.77*** (1.45, 2.17)
	[28d, 1y)	404	9,288.69	638	15,074.35	1.03 (0.91, 1.17)	1.01 (0.89, 1.15)	1.06 (0.92, 1.21)	1.05 (0.92, 1.21)
	[1y, 5y)	393	33,711.11	611	50,957.39	0.99 (0.87, 1.12)	1.00 (0.88, 1.14)	0.99 (0.87, 1.13)	0.98 (0.86, 1.12)
	[0, 5y)	1007	43,827.27	1450	67,387.50	1.10* (1.02, 1.20)	1.11* (1.02, 1.21)	1.13** (1.03, 1.23)	1.12** (1.03, 1.22)
Somkhele	[0, 28d)	23	142.72	23	209.00	1.46 (0.82, 2.61)	1.36 (0.74, 2.48)	1.38 (0.75, 2.53)	1.40 (0.76, 2.58)
	[28d, 1y)	97	1,563.59	109	2,323.72	1.32* (1.00, 1.73)	1.48** (1.11, 1.98)	1.51** (1.12, 2.05)	1.52** (1.13, 2.04)
	[1y, 5y)	37	5,527.79	68	8,157.08	0.81 (0.54, 1.21)	0.91 (0.59, 1.38)	0.83 (0.55, 1.26)	0.83 (0.55, 1.26)
	[0, 5y)	157	7,234.10	200	10,689.79	1.16 (0.94, 1.43)	1.28* (1.03, 1.59)	1.27* (1.01, 1.59)	1.28* (1.02, 1.60)

* *p* < 0.05;

** *p* < 0.01;

*** *p* < 0.001.

*Note*: d refers to days; y refers to years.

*Source*: Authors’ analysis of data from HDSS sites in Basse [2011, 2015), Siaya [2010, 2014), and Somkhele [2001, 2004).
